# Novelties in treatment of locally advanced rectal cancer

**DOI:** 10.12688/f1000research.16194.1

**Published:** 2018-11-29

**Authors:** Fabian Grass, Kellie Mathis

**Affiliations:** 1 Department of Surgery, Division of Colon and Rectal Surgery, Mayo Clinic, Rochester, MN, USA

**Keywords:** Rectal cancer, treatment, surgery, neoadjuvant, watch and wait

## Abstract

Treatment of locally advanced rectal cancer is evolving through surgical innovation and paradigm shifts in neoadjuvant treatment. Whereas local recurrence was a significant concern before the systematic implementation of neoadjuvant chemoradiation therapy and surgery according to total mesorectal excision principles, distant relapse remains a major drawback. Hence, efforts in recent years have focused on delivering preoperative chemotherapy regimens to overcome compliance issues with adjuvant administration. In parallel, new surgical techniques, including transanal video-assisted total mesorectal excision and robot-assisted surgery, emerged to face the challenge to navigate in the deep and narrow spaces of the pelvis. Furthermore, patients experiencing a complete response after neoadjuvant treatment might even escape surgery within a close surveillance strategy. This novel “watch and wait” concept has gained interest to improve quality of life in highly selected patients. This review summarizes recent evidence and controversies and provides an overview on timely and innovative aspects in the treatment of locally advanced rectal cancer.

## Introduction

During the last decades, new surgical and medical treatment strategies for stage II and III locally advanced rectal cancer (LARC) emerged and challenged classic treatment schemes ^1^. Laparoscopic surgery according to the principles of total mesorectal excision (TME) is now widely established as standard in well-resourced countries. However, this approach is technically demanding for low-rectal cancers in patients with unfavorable baseline conditions and narrow pelvises. In recent years, robot-assisted surgery and the transanal video-assisted approach (TaTME) have been described as modern alternatives for overcoming these difficulties ^2, 3^.

In parallel, the standard concept of neoadjuvant fractioned long-course chemoradiation therapy (CRT) followed by surgical resection six weeks later and adjuvant chemotherapy has been questioned ^4^. Distant relapse has become more of a concern than local recurrence, which is significantly better controlled since the systematic implementation of preoperative chemoradiation and surgery carried out according to the TME principles ^5^. On the other hand, adjuvant systemic treatments may be delayed or even omitted in up to 50% of patients as a consequence of major surgery with potential complications, slow recovery, and interference with loop ileostomy reversal ^6– 8^; hence, administration of systemic chemotherapy before the actual surgical resection has been suggested to overcome these drawbacks ^9, 10^.

One of the most debated treatment concepts today is the “watch and wait” approach, which implies a non-operative surveillance strategy in patients with complete clinical response to neoadjuvant treatment ^11^. This approach notably gained interest after observing equivalent long-term results in patients with clinical stage 0 disease undergoing surgery as compared with patients within a close surveillance strategy ^12^. In this review, recent developments and controversies in surgical management, neoadjuvant strategies, and non-operative management of LARC are summarized and discussed.

## Surgical innovations

### Laparoscopic surgery

Laparoscopy has been established as the standard approach for colorectal resections in most developed countries because of advantages regarding postoperative pain, return of bowel function, and length of hospital stay without compromising oncological safety ^13– 15^. Particularly for rectal cancer, the plane of surgery has been identified as an important predictive factor of local recurrence ^16^, and principles of TME with sharp dissection of the mesorectal fascia are now considered standard ^17, 18^. However, laparoscopic dissection of a deep-situated LARC might be challenging and has raised concerns regarding long-term oncologic outcome. Several randomized controlled trials compared oncological outcomes between open and laparoscopic resections. The large COLOR II trial, which randomly assigned 1044 patients, found no difference in locoregional recurrence at three years, which was 5% in both groups ^19^. One interesting finding was that circumferential resection margins (CRMs) were more often compromised in the laparoscopic group for mid-rectal cancers, whereas for low-rectal cancers, CRM was more often positive after open surgery. This latter finding, however, was explained by the particular challenge of abdominoperineal resections. The COREAN trial, which looked at three-year disease-free survival in mid- and low-rectal cancers, found similar outcomes, thus justifying a wide adoption of the laparoscopic approach ^20^. However, heterogeneity of endpoints as well as differences in disease stages, tumor location, and neoadjuvant chemotherapy impede uncritical comparisons of the results of these studies and potentially lead to misinterpretations. On the other hand, two other randomized trials, published in 2015, raised concerns about the safety of a laparoscopic approach for LARC since they failed to demonstrate non-inferiority of the laparoscopic approach using pathological endpoints ^21, 22^. The first was a multicenter North American study that included only experienced, credentialed surgeons; a composite pathological endpoint to determine surgical quality was chosen ^21^. The second was a similarly designed Australasian trial that reproduced these results ^22^. As a common conclusion of the trials, routine use of the laparoscopic approach could not be recommended beyond doubt. However, all trials, including the formerly conducted CLASICC trial, acknowledged advantages of the laparoscopic approach regarding improved surgical and functional recovery and decreased length of stay ^23– 25^.

In summary, despite favorable short-term outcomes, ambiguous results regarding oncological long-term outcome after laparoscopy probably emphasize the importance of surgical expertise and dexterity when choosing one approach over another. Long-term oncologic results of the North American and Australasian trials are anxiously awaited.

### Transanal total mesorectal excision

Low LARC needing sphincter-sparing resection can be challenging because of narrow pelvic anatomy, especially in male patients and in patients with a high body mass index and fatty mesorectum ^26^. Notably, clean distal resection margins can be difficult to achieve because of visibility issues ^27^. To overcome these challenges, a new approach has been described in recent years, taking advantage of the magnification of a laparoscope to allow better visualization of the lower mesorectum and endangered structures through the anus. A recent meta-analysis of seven studies showed encouraging outcomes regarding completeness of specimens and postoperative complications. However, individual study quality was modest (retrospective case control trials), the approach was barely standardized, and all studies were conducted in high-volume expert centers. Hence, confirmation of these results in less experienced centers might not be achievable right away. Two randomized trials of the GRECCAR and COLOR study groups are recruiting patients ^28^. Furthermore, a recent expert consensus statement provided guidance for optimal clinical practice ^29^. Several questions remain unanswered, in particular whether a one- or two-team approach is preferable and to what extent surgical experience and proper training are required to overcome the significant learning curve ^30, 31^. Obviously, at this stage, long-term functional and oncological outcomes are not yet available.

### Robotic surgery

The robotic platform da Vinci (Intuitive Surgical, Sunnyvale, CA, USA) entered the market in 2001 and was applied mainly for urologic and gynecologic surgery. Even though robot-assisted laparoscopic prostatectomy was not superior to open retropubic prostatectomy regarding short-term postoperative outcomes in a recent randomized controlled phase 3 study ^32^, the robotic approach is widely established in the urologic field in well-resourced countries ^33^. It was estimated that up to 80% of radical prostatectomies will be performed robotically in the US by 2020 ^34^.

In recent years, robot-assisted surgery gained interest for rectal cancer resections because of its optimal visualization and improved navigation in the narrow pelvic space. However, meta-analyses did not show any advantage over laparoscopic surgery, except for decreased conversion rates ^2^. Mainly retrospective case series demonstrated similar completeness of TME, similar rates of CRM positivity, and equal short-term oncological outcomes ^35, 36^, whereas a smaller study suggested improved quality of the specimen after robotic TME ^37^. The most important study in the field was published recently: The ROLARR study, an international multicenter prospective trial, randomly assigned 471 patients to either conventional laparoscopic or robotic-assisted resections ^38^. The study failed to demonstrate significant benefits of robotic surgery regarding the main outcomes of CRM positivity, TME quality, intra- and postoperative complications, and 30-day mortality. However, the wide range of experience among operating surgeons was criticized.

In conclusion, it probably comes down to a matter of experience and dexterity with either approach, and no clear recommendation can be made based on the available evidence.

## Neoadjuvant strategies

The guidelines of the National Comprehensive Cancer Network (NCCN) for LARC recommend a multidisciplinary approach with neoadjuvant CRT, surgery according to TME principles, and adjuvant chemotherapy ^39^. Neoadjuvant CRT was defined as standard mainly because of its potential to decrease 5- and 10-year pelvic recurrence rates ^40^. However, whether long- or short-course radiotherapy is preferable remains matter of debate; in the US, the overwhelming majority of radiation oncologists still favor long-course CRT ^41^. Classically, preoperative chemotherapeutic agents act as radiosensitizers. Although 5-fluorouracil (5-FU) is widely accepted, the oral 5-FU prodrug capecitabine was identified as a valid treatment alternative more recently ^42^.

### Neoadjuvant chemotherapy

The concept of neoadjuvant CRT was re-evaluated recently. Although local recurrence was better controlled, facing the estimated five-year distal relapse rate of 35% became the primary target ^43^. Administration of systemic chemotherapy in the neoadjuvant setting, before or after CRT, gained interest to face the drawback of low compliance in the adjuvant setting ^6, 44^. Neoadjuvant chemotherapy further allows timely identification of non-responders and treatment of occult micro-metastases several months preoperatively ^4, 45^. A wide range of drugs, including oxaliplatin as an adjunct to CRT, failed to demonstrate clear benefits in several high-quality studies and this was due mainly to increased toxicity ^46, 47^. However, more recent data showed improved disease-free survival when adding oxaliplatin to both preoperative CRT and postoperative chemotherapy ^48^. Several further phase II trials showed similar promising results without jeopardizing planned CRT or increasing surgical complications ^9, 49– 51^, labelling the concept of *total* neoadjuvant chemotherapy as safe and feasible. Splitting of adjuvant chemotherapy by delivering at least some cycles before CRT and the remaining post-surgery has also been described as an alternative ^52, 53^. A randomized trial in North America (NRG GI002) is accruing patients for a total neoadjuvant approach.

Despite these encouraging results, systemic chemotherapy is still administered primarily in the adjuvant setting. Fluoropyrimidine-based regimens for four months are recommended by consensus guidelines, even though the value of the regimens was debated because of incongruent results when administered to all patients, independent of pathologic tumor stage ^54, 55^.

### Selective preoperative radiotherapy

Given the drawback of long-term morbidity with pelvic irradiation and widespread application of TME principles to decrease local recurrence, a subset of patients may be eligible to avoid preoperative radiation and to undergo solely neoadjuvant systemic chemotherapy. However, only relatively small single-arm studies are available to date and results are promising: radiographic down-staging was achieved in 25 to 70% of patients, and local recurrence rates were not increased in these highly selected patients ^56– 58^. Large studies are ongoing ^44^ and today this approach is used primarily in trial settings. The randomized phase III PROSPECT (Preoperative Radiation or Selective Preoperative Radiation and Evaluation Before Chemotherapy and TME) trial is assessing this strategy in patients with uncompromised CRM (ClinicalTrials.gov Identifier: NCT01515787).

## Watch and wait

Surgery for LARC has a significant impact on the patient, and severe complications occur in up to 22% of patients ^21^. Quality of life might be significantly impaired postoperatively, especially since sphincter preservation is possible in only 50% of patients with low-rectal cancer ^59^. Organ-preserving strategies thus gained interest in patients with a complete clinical response to neoadjuvant treatment ^12^. The most critical aspect when considering organ preservation is the accuracy in assessing tumor response. Optimal timing of the assessment is crucial since tumor regression after CRT appears to be time-dependent. Although non-responding tumors should be re-assessed within six to eight weeks, responders might benefit from at least a 12-week interval, according to a Brazilian pioneer group of this approach ^60^. A large British study assessed oncological outcomes in 259 patients with clinical complete response to CRT through a propensity score–matched cohort analysis. No difference in three-year disease-free survival was noted, and permanent colostomy could be avoided in 74% of patients in the surveillance group ^61^. A recent systematic review and pooled analysis described an overall complete clinical response rate to neoadjuvant CRT of 22.4% ^62^. Seventeen studies reported on 692 patients with complete clinical response and were retained in this systematic review; most studies were relatively small retrospective cohort studies. Whereas 68% of patients presented with cT3 tumors, 50% were node-positive on pre-treatment staging. The time to response assessment varied widely, from 3 to 24 weeks. Most studies (67%), however, re-assessed treatment response after a minimum of eight weeks. Treatment response was assessed through triple assessment (digital rectal examination, endoscopic, and radiological) in 88% of studies. Tumor regrowth within the three-year observation period was noted in 22.1% of patients and 68% of these patients relapsed during the first year of surveillance.

Taken together, the vast majority of patients who received current neoadjuvant CRT did not achieve a complete response. In recent years, intense molecular biology research aimed to identify reliable biomarkers to predict complete response. Several recent studies described extramural vascular invasion as a promising prognostic factor on high-resolution magnetic resonance imaging (MRI) to assess risks of metastatic disease after CRT ^63– 65^. Furthermore, MRI-based texture parameters have been investigated more recently as potential predictors of long-term survival in LARC ^66, 67^. Accuracy of re-assessment of tumor response is of utmost importance. A recent meta-analysis described a sensitivity of 77% and a specificity of 94% of MRI in predicting CRM involvement ^68^. The highly cited MERCURY trial found that MRI involvement of the CRM was the only preoperative staging parameter for predicting local recurrence and survival ^69^. Thus, high-resolution MRI has become an indispensable tool to confirm clinical and endoscopic findings of a complete clinical response. Patients within a watch-and-wait strategy should adhere to a strict follow-up program, ideally with a prospective registry, to allow successful salvage surgery if deemed necessary ^70^.

## Further considerations and perspectives

A particular challenge represents the management of LARC in frail patients and the elderly. Preoperative frailty and nutritional assessment together with quality-of-life considerations are of utmost importance when defining surgical and oncologic strategies ^71, 72^. Importantly, radical surgery should be considered only after careful case-by-case evaluation involving caregivers, patients, and their family through shared decision making to aim for the best possible outcomes *and* quality of life ^73– 75^.

Promising results can be expected from studies focusing on disease monitoring by genomics, such as target sequencing of circulating tumor DNA ^76, 77^ or apoptosis-related genes, including the p53-signaling pathway ^78, 79^. Findings of these innovative research fields will likely further impact treatment strategies of LARC in the future.

## Conclusions

Classic concepts of neoadjuvant and surgical treatment for LARC are being challenged. Modern surgical concepts aim to facilitate surgical navigation in the pelvis, but the ideal approach has not yet been described. In particular, robotic surgery has not yet provided the expected breakthrough.

An individualized oncological approach for selected patients is about to replace the classic tri-modality treatment scheme, according to numerous high-quality multicenter randomized trials, and several ongoing multicenter trials may liven the debate for years to come.

## Abbreviations

5-FU, 5-fluorouracil; CRM, circumferential resection margin; CRT, chemoradiation therapy; LARC, locally advanced rectal cancer; MRI, magnetic resonance imaging; TME, total mesorectal excision
